# The lie of pandemic pivot and essential work

**DOI:** 10.1177/1473325020973394

**Published:** 2021-03

**Authors:** Alexandra Crampton

**Affiliations:** Department of Social and Cultural Sciences, Marquette University, Milwaukee, WI, USA

**Keywords:** Social policy, critical reflection, social justice, social intervention, crisis response, Covid-19

## Abstract

My emotional responses to this moment include feelings of anger, hope, and déjà vu. Although the scope and scale of this pandemic is unprecedented in our lifetimes, what has been especially hard is not necessarily new – nor entirely unprecedented – and therefore unavoidable. In this essay, I reflect on what was avoidable and call for better response. We must question the seemingly benign (if not optimistic) terms emerging as pandemic discourse, such as “pivot to a new normal” and “essential work,” for what they reveal of social injustice and failure to avert future crisis.

Until now, I did not know how hope and anger can be so easily intertwined. My sense of hope derives from what moral philosopher Michael Walzer would call the “thinness” of this moment (Walzer 1994). In contrast to emphasis on thickness, as in “thick description” of qualitative scholarship, there are moments in which the depth and importance lie in how this complexity collapses into shared experience. His example, from 1989, was back when people in Prague were protesting life under the former Soviet Union with signs that simply stated, “Truth” and “Justice.” He felt kinship with them, and felt deep connection to the meaning of these words, even though he had never traveled to that part of the world, nor studied its culture. As he stated, he could “March in other peoples’ parades” (and protests). We seem to keep having such moments during these first four months of global pandemic.

Anthony Appiah (2006) draws from Walzer’s term in his book, *Cosmopolitanism*. A central question in the book is how we can co-exist globally. As a philosopher, Appiah has many ideas but foregos clearly proposed courses of action. A virus suddenly prodded us for these proposals by bringing a halt to life as we had known it, exposing the fragility of our health and economic infrastructures, and threatening “bare life” (Agamben 1998) as well as the normalcy of our social lives. Despite not being ready, despite lack of evidence-based-pandemic-practice (EBPP), we have had to seek answers.

Unfortunately, the panic of this moment can mean less seeking and more reacting. And, seeking itself should not have to await crisis for serious attention. Taking a longer-term view, the science of infectious disease is not the problem as much as overcoming human barriers to implementation: In science, we have encountered infectious disease challenges before, have learned how to develop vaccines, and have provided care that greatly improves chances of survival. However, underlying infrastructures for basic research, disease prevention, and coordinated response have been eroded or simply absent. As a qualitative researcher, I wonder if the reason why Covid-19 seems unusually wily and unwieldy is simply because scientists and policymakers must confront this pandemic in real time, as the world watches the messiness of preliminary work and intervention implementation. In qualitative field work, this *is* my normal; I am constantly navigating gaps between expectations based on policy intentions and real-time encounters amidst the messiness and complexity of lives disrupted through poverty, racism, and other vulnerabilities (e.g. Crampton 2016). Now the tension of this challenge, between expertise and lived chaos, is on display as scientists and policymakers scramble to propose and implement clear and effective interventions.

A leading epidemiologist has recently advised that, “We need an epidemic of kindness” to combat the overwhelming uncertainty and challenge of this time ( Osterholm 2020). This statement fills me with hope and rage because we already know this in social work. I study social interventions that defy such facile answers as search for a single cure or course of therapy. And yet, qualitative social work simply is not given the same status or attention as promises to cause the desired effect for the most people and in the least time. We already know how people’s health and well-being is contextual and at-risk due to human systems of social inequality as well as overt hostility to people of color and people in poverty. We have been trying to raise the importance of this knowledge and the slow, dedicated work it requires. We should be at the forefront in this pandemic as experts on what could have been done and ought to be done to avert the next disaster. Instead, the focus from most experts follows from pressure to provide a time-limited plan to end the pandemic and get back to “normal.” Yes, of course, what else is there to say when crisis has not been averted but to call for an epidemic of kindness?

## A local view of a global déjà vu

Recent news coverage of racial health disparities exposed through Covid-19 brings to me a sense of déjà vu. Residing in the hyper-segregated city of Milwaukee, Wisconsin, where one’s postal code is linked to disparate health and social outcomes, I see precedents of the pandemic inequities daily. In privileged postal codes, disruption, crisis, and chaos are constructed as rare and rarely linked to systemic problems. By associating most of Milwaukee’s problems with minoritized people and unusual situations, the resulting social construction is that risk itself is rare and avoidable through privilege, especially white privilege. Across the city, this helps the sufferings of health and economic crises to be kept discursively small—as local and personal problems. This enables policymakers and nonprofit grant funders to imagine social problems as manageable and dislocated from more general policy concerns of society and economy, such as the interplay between economic and welfare policies. There seems to be little cause for deeper attention to how fragile life has become within systems of segregation and divided attention.

And then came a virus. Who knew that such a tiny interlocutor could burst through socially constructed binaries of have/have-nots, and of privilege/risk? Suffering, it turns out, can come for us all. An encouraging response to shared suffering is the countless examples of compassion and giving. In this moment, millions and perhaps billions of strangers suddenly feel kinship as, “mutuality of being” (Sahlins 2013). Such kinship especially awakens through suffering brought by Covid-19 deaths. I felt this acutely in learning how a friend’s brother suddenly died of a heart attack after, “he was doing so well” through the incredible stress of ventilated life in an ICU for two weeks. Shared suffering due to Covid-19 can intertwine compassion with rage in recognition of “stupid deaths” (Farmer 2011), which are those deaths that could have been avoidable if only investments in health and health care infrastructures had been made . These stupid deaths bring home the message of global – and not merely national – challenge as this suffering transcends political borders. This message then can circle us back to hope; prior to this moment, it seemed impossible to conjure the political will for even raising such issues. Now, compassionate response to viral suffering shows how boundaries between those at risk and those protected can collapse, potentially igniting the energy to overcome political resistance to systemic change.

However, current social intervention discourse suggests that this may not happen. Consider, for example, the phrase, “pivot to a new normal.” This phrase is used to assure those of us for whom crisis is unusual that we can return to our normal, albeit not entirely. The phrase also lies in marketing and policy efforts to convince that nothing fundamental must change in markets and consumption. Technology is a key player, as we learn to resume previous activities with the help of Zoom. With attention on what we can do to resume what some expect as “normal,” there is a danger of losing sight of how “normal” for so many has meant disruption, chaos, and crisis. Ironically, successful pivot threatens the primacy of this moment’s moral thinness, in which we collectively recognize how easily we can each be made fragile in our modern economy. The pivot we truly need is to pivot on this feeling, this basis for compassion. As the good doctor remarked, we ought to ask how to better care for each other. Then, we must scale up from interpersonal levels to that of public policy. We should not seek return to a “normal” that divides people into have and have-nots.

## The lie of essential work

In addition to the insidious implications of pivot, we ought to examine more deeply the policy term, “essential work” (also phrased as “critical work”). The pandemic brought a sudden halt to many people’s assumptions about their agency in choosing work. Suddenly, those who were “essential” and “not essential” could be told whether to work or stay at home, and under what conditions. Again, a temporary collapsing of worlds (and postal codes) in which people all-too-familiar with public assistance are temporarily joined by the “mainstream.” The surface level reason for quarantine-oriented safety orders, such as “stay-at-home,” was to protect individual and public health. In practice, categorizing work as “essential” or not has led to two categories of risk; those whose work is “essential” and thus requires their labor despite health risks, and those whose work is nonessential and thus requires loss of vital income. For low wage workers, this forced choice between health and income is neither unprecedented nor avoidable. In the U.S., what may surprise these workers is the size of cash assistance: A US$1200 stimulus check, followed by a self-congratulatory reminder from the president, seems remarkable given sustained efforts over decades to downsize and stigmatize welfare state assistance. A good, contrasting example is the cash assistance program called Temporary Assistance to Needy Family (TANF). Historically, TANF began as “mother’s pensions,” given to Civil War veteran widows in recognition that raising children requires labor, which in turn requires support (Gillon 2000). Today, of course, such stay-at-home decisions are scorned, leaving both parents pressured to work while paying the extra bill of private child care.

Another problem with the term “essential” is that it implies value and yet is used to eliminate. In practice, the policy results in eliminating as many active people and the support they need as possible. We are now back to déjà vu as this policy has already been at work in the downsizing of social services to eliminate more extensive engagement with clients, and in scrambling for funding to conduct qualitative social work. In social work, we lose funding for proactive, community-building interventions because reactive programs that address immediate crises are more highly valued (greater imagined Return-on-Investment (ROI)). In addition, limited funding pitches people in different social work areas against each other, sometimes reducing us to arguing which of “our” populations is the most vulnerable, and therefore the most deserving of precious funds. Yet, as the spread of infectious disease shows us, these “siloed” areas of social work artificially divide very interdependent populations. The thinking underlying “essential” effectively divides where division can distort understanding, cause unnecessary competition, and lead to less holistic intervention approaches.

We should ask now how social work research and practice could change if the costs of “essential” work downsizing were factored into grant competitions and social service funding. The greater humility of qualitative social work calls for asking for things that have been constructed as nonessential; such as more time for immersion in local contexts, coupled with more openness to direction from local people as a way to ensure that the research is relevant to their needs. The path from problem to solution is often nonlinear, uncertain, and fraught – this reality cannot be simply eliminated because it seems inefficient or too slow. Then, turning to the pandemic response reveals parallel problems; for example, how simply announcing rules without more careful cultivation of rule-making and implementation has led to widespread variation and protest rather than collective effort to weather this storm together. And, such collective weathering should not have awaited crisis for inspiration; this consciousness must be cultivated through relationship building. Without *this* essential work, we are left in cycles of elimination followed by expensive repair packages. We ought to ask, which approach costs more in human and financial terms? What if the moral weight of public policy, a sort of compassion dividend, becomes part of a cost/essential equation?

## Reclaiming social work as “essential” through compassionate pivot

The next question is, “So what?” and why would anyone listen to us? I suggest that the panic of this moment, and the widespread felt experience of injustice, insecurity, and search for hope are opportunities for us in social work to reclaim the meaning of “essential.” Empathy can be a powerful motivator, and privileged people worldwide are undergoing a crash course in threat to basic health and well-being. We must urge those who enjoyed the earlier “normal” access to health and good income (with benefits) to reflect on what sudden loss of access feels like, and how this experience is not unprecedented for many in our country, and our world. We can teach a pandemic pedagogy that links Covid-19 disruptions to the lesson that no one, ultimately, is immune to suffering. We can ask how much of the suffering of this moment *was avoidable*, and ask how much cost for relief has been incurred through previous elimination of what were deemed nonessential resources and services.

We should be afraid – but not of a microbe. We ought to fear the fractures and fragility of our economic, health and welfare systems that are so easily exposed by a tiny interlocutor. We ought to challenge how cost is calculated in local and global economies—and compare these with the high cost of crisis “relief” packages. In doing this, we reclaim the term “essential” and call for “pivot” to a new level of compassion-driven prevention and greater crisis preparation. This includes accepting the “cost” of such qualitative social work practices as taking time, considering context, and ensuring agency. Otherwise, we risk pivot back to “normal.” This merely brings us yet deeper into socioeconomic disparities in which a comparatively few will simply shift from a sense of entitlement to gratitude for the privilege of social protection and return to economic security.

## Conclusion

The sufferings of this time are an importantly thin moment. And, what is happening now is not entirely new, unprecedented, and unavoidable. This means it can happen again. In social work, we ought to hold to this “thin” moment and resist “pivot to a new normal.” For many in the U.S. and abroad, it has been “normal” to be in crisis. The difference, for them, is more in in how high the waters have risen rather than that they have risen at all. As a teachable moment, we should highlight what was a natural, even expected, outcome from socio-economic trends. We need systemic change to avoid “stupid deaths.” A qualitative social work perspective is uniquely poised in this effort due to our countervailing tendencies to emphasize what has been deemed as not essential, such as the slow work of deeper engagement and reflection that this special issue offers. Through these engagements, we ought to reclaim the term “essential” in advocating for change that might help us to better “pivot” in response to the next “unprecedented crisis,” or, to modify a title from James Baldwin (1963), the virus next time.
